# Fusobacterium nucleatum Metabolically Integrates Commensals and Pathogens in Oral Biofilms

**DOI:** 10.1128/msystems.00170-22

**Published:** 2022-07-19

**Authors:** Akito Sakanaka, Masae Kuboniwa, Shuichi Shimma, Samar A. Alghamdi, Shota Mayumi, Richard J. Lamont, Eiichiro Fukusaki, Atsuo Amano

**Affiliations:** a Department of Preventive Dentistry, Graduate School of Dentistry, Osaka University, Osaka, Japan; b Department of Biotechnology, Graduate School of Engineering, Osaka University, Osaka, Japan; c Department of Oral Immunology and Infectious Diseases, School of Dentistry, University of Louisville, Louisville, Kentucky, USA; Arizona State University

**Keywords:** *Fusobacterium nucleatum*, *Porphyromonas gingivalis*, arginine deiminase system, metabolic cross-feeding, oral biofilms, periodontitis, polyamines

## Abstract

Fusobacterium nucleatum is a common constituent of the oral microbiota in both periodontal health and disease. Previously, we discovered ornithine cross-feeding between F. nucleatum and Streptococcus gordonii, where S. gordonii secretes ornithine via an arginine-ornithine antiporter (ArcD), which in turn supports the growth and biofilm development of F. nucleatum; however, broader metabolic aspects of F. nucleatum within polymicrobial communities and their impact on periodontal pathogenesis have not been addressed. Here, we show that when cocultured with S. gordonii, F. nucleatum increased amino acid availability to enhance the production of butyrate and putrescine, a polyamine produced by ornithine decarboxylation. Coculture with Veillonella parvula, another common inhabitant of the oral microbiota, also increased lysine availability, promoting cadaverine production by F. nucleatum. We confirmed that ArcD-dependent S. gordonii-excreted ornithine induces synergistic putrescine production, and mass spectrometry imaging revealed that this metabolic capability creates a putrescine-rich microenvironment on the surface of F. nucleatum biofilms. We further demonstrated that polyamines caused significant changes in the biofilm phenotype of a periodontal pathogen, Porphyromonas gingivalis, with putrescine accelerating the biofilm life cycle of maturation and dispersal. This phenomenon was also observed with putrescine derived from S. gordonii-F. nucleatum coculture. Lastly, analysis of plaque samples revealed cooccurrence of P. gingivalis with genetic modules for putrescine production by S. gordonii and F. nucleatum. Overall, our results highlight the ability of F. nucleatum to induce synergistic polyamine production within multispecies consortia and provide insight into how the trophic web in oral biofilm ecosystems can eventually shape disease-associated communities.

**IMPORTANCE** Periodontitis is caused by a pathogenic shift in subgingival biofilm ecosystems, which is accompanied by alterations in microbiome composition and function, including changes in the metabolic activity of the biofilm, which comprises multiple commensals and pathogens. While Fusobacterium nucleatum is a common constituent of the supra- and subgingival biofilms, its metabolic integration within polymicrobial communities and the impact on periodontal pathogenesis are poorly understood. Here, we report that amino acids supplied by other commensal bacteria induce polyamine production by F. nucleatum, creating polyamine-rich microenvironments. Polyamines reportedly have diverse functions in bacterial physiology and possible involvement in periodontal pathogenesis. We show that the F. nucleatum-integrated trophic network yielding putrescine from arginine through ornithine accelerates the biofilm life cycle of Porphyromonas gingivalis, a periodontal pathogen, from the planktonic state through biofilm formation to dispersal. This work provides insight into how cooperative metabolism within oral biofilms can tip the balance toward periodontitis.

## INTRODUCTION

Periodontitis is a multifactorial chronic disease with diverse phenotypes often characterized by inflammatory destruction of periodontal tissues (1). The risk and severity of periodontitis are attributed to a dysbiotic transition in the community of microbes residing in the subgingival biofilm (2). In this process, Porphyromonas gingivalis plays a central role, although recent studies suggest that colonization of P. gingivalis does not necessarily elicit disease and that full virulence requires the presence of the commensal microbiota, highlighting the importance of polymicrobial synergy in the disease etiology (3). Notably, a recent metatranscriptomic analysis of subgingival plaque from periodontitis patients showed highly conserved metabolic profiles, even though substantial microbiome variation was observed (4). This finding suggests that the transition between periodontal health and disease is more correlated with a shift in metabolic function of the community as a whole, rather than with the presence of individual taxa, drawing attention to metabolic aspects of microbial communities in periodontal pathogenesis.

Metabolic cross-feeding is one of the key factors directing the establishment of a community and the metabolism therein (5). A subset of oral streptococci engages in cross-feeding interactions with other community members that often result in elevated pathogenicity of microbial communities (6). A well-known example is lactate cross-feeding from Streptococcus gordonii to lactate-utilizing bacteria, such as Veillonella parvula and Aggregatibacter actinomycetemcomitans, where S. gordonii releases lactate as an end product of glucose metabolism, thus allowing complementary utilization of available glucose and promoting fitness of these organisms in the community (7, 8). S. gordonii also impacts the pathogenicity of P. gingivalis through the secretion of para-aminobenzoic acid, which promotes *in vivo* fitness and colonization of P. gingivalis, albeit with diminished virulence (9). Additionally, *V. parvula* produces a soluble molecule that supports the growth of P. gingivalis from small populations, enabling *in vivo* colonization and virulence (10). Given recent bioinformatic research showing that the oral microbiome can produce an enormous number of small metabolites that may influence oral pathophysiology (11), many more metabolic interactions between oral microbes likely remain to be discovered.

Fusobacterium nucleatum has been implicated in both periodontal health and disease due to its frequent detection in subgingival plaque samples of both healthy and diseased sites (12–14). While this species is well known for its organizing role in oral biofilms through the expression of multiple adhesins, whereby it can direct the spatial relationships among early and later colonizers (15), metabolic aspects of interspecies interactions between F. nucleatum and other community members remain relatively unknown compared to physical ones. Earlier studies showed that F. nucleatum supports the growth of P. gingivalis by rendering the microenvironment alkaline and less oxidative (16). F. nucleatum has a preference for peptides and amino acids and produces butyrate and ammonia as end products of the fermentation pathways, starting mainly from glutamate and lysine (17, 18). The aforementioned metatranscriptomic analyses showed that despite nearly the same abundance of F. nucleatum between healthy and periodontitis samples, its metabolism is markedly changed under those two conditions (4). Considering that F. nucleatum is also strongly linked to serious systemic conditions such as adverse pregnancy outcomes and colorectal cancer (14), it is important to improve our basic understanding of the metabolic properties of F. nucleatum within polymicrobial communities.

Recently, we identified a novel metabolic interaction between S. gordonii and F. nucleatum (19), starting from the metabolism of arginine by S. gordonii as a substrate in the arginine deiminase system (ADS), through which arginine is converted to ornithine with concomitant production of ammonia and ATP. An arginine-ornithine antiporter of S. gordonii, ArcD, then excretes ornithine as a metabolic by-product of the ADS, which in turn enhances the growth and biofilm development of F. nucleatum. However, it is unknown how ornithine influences F. nucleatum metabolism and what consequences this interaction has for disease etiology. Therefore, in this study, we set out to further dissect the metabolic interactions mediated by F. nucleatum within multispecies consortia and determine whether the engagement of F. nucleatum in metabolic interactions in oral biofilms can impact the potential pathogenicity of the microbial community. By using a synthetic community model as well as clinical plaque samples, this study supports an emerging role of F. nucleatum as a metabolic bridge to relay the metabolic flow between initial and late colonizers, thereby creating favorable conditions for the outgrowth and spread of P. gingivalis.

## RESULTS

### Distinct metabolic profiles in F. nucleatum cocultured with S. gordonii and/or *V. parvula*.

We performed untargeted analysis of the intra- and extracellular metabolite changes in F. nucleatum when cocultured with S. gordonii and/or another common inhabitant of the oral microbiota, *V. parvula*. To focus upon metabolic aspects of interspecies interactions, we used Transwell assays, which physically separate bacterial populations but allow for metabolite exchange via a shared medium reservoir. The system was anaerobically incubated in triplicate for 6 h in chemically defined medium (CDM) with ammonium sulfate as the sole nitrogen source, so that limiting exogenous nutrients could facilitate interspecies nutritional interactions in the community. Overall, we identified 111 extracellular and 85 intracellular metabolites, 52 of which were detected both intra- and extracellularly (see Data Sets S1 and S2 in the supplemental material).

**FIG 1:**
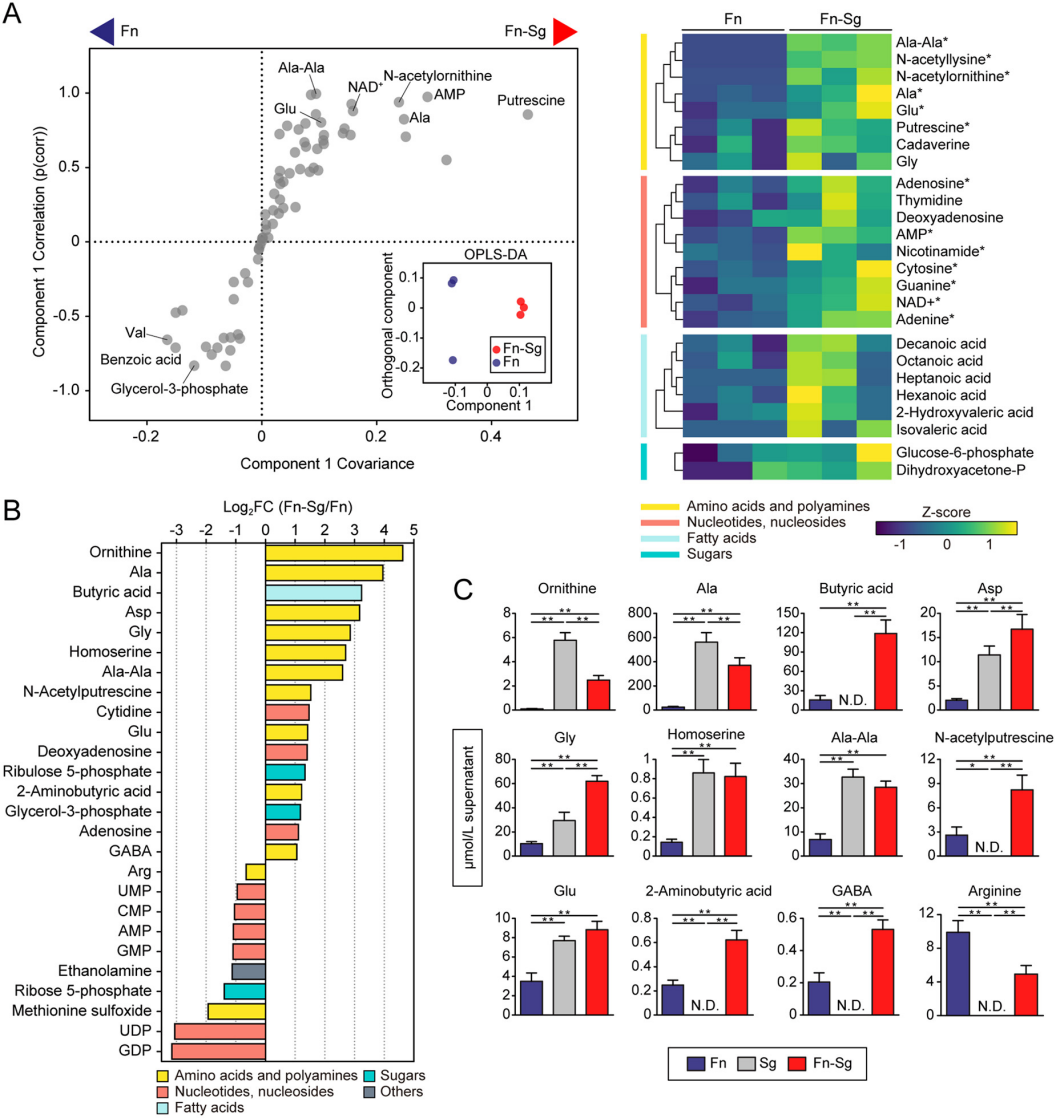
Intra- and extracellular metabolite changes of F. nucleatum cocultured with S. gordonii. (A) Intracellular metabolite changes in F. nucleatum cocultured with S. gordonii. A total of 1.4 × 10^10^ cells of F. nucleatum were anaerobically cultured in CDM in the lower chamber of Transwell plates with membrane inserts, into which 1.4 × 10^10^ cells of S. gordonii in CDM or an equal volume of fresh CDM (as a control) were added. After 6 h, F. nucleatum cells were harvested, and metabolic profiles were analyzed by CE-TOFMS. The OPLS-DA S-plot and score plot (inset) are shown in the left panel, where metabolites toward both sides of the S-shape distribution represent metabolites that characterize each group with high reliability; putrescine was among the most impacted metabolites in cocultures. The right panel shows a clustered heatmap of intracellular metabolites with high reliability in the S-plot (p[corr] > 0.6). Columns represent biological replicates of F. nucleatum samples, grouped by culture method, and rows represent individual metabolites, whose levels are displayed as Z scores. Asterisks denote significant differences in univariate methods. *, *P* < 0.05 (Mann-Whitney U test). (B) Extracellular metabolites displaying a concentration change in cocultures compared to monocultures (log_2_ fold change [FC] < −0.6, log_2_ FC > 1, and *P* < 0.05; Mann-Whitney U test). (C) Levels of the selected metabolites in spent media of cocultures and each monocultures, determined by UPLC. For this, the same procedures were repeated with the additional control of S. gordonii monocultures. Error bars correspond to standard deviations. *, *P* < 0.05; **, *P* < 0.01 (one-way ANOVA with Tukey’s test). Fn, F. nucleatum subsp. *nucleatum* ATCC 25586; Sg, S. gordonii DL1.

In coculture with S. gordonii, orthogonal projection to latent structure-discriminant analysis (OPLS-DA), a multivariate statistical method suitable for identification of subsets of variables for discrimination between groups, revealed that the intracellular metabolic profile of F. nucleatum clustered distinctly from that of F. nucleatum alone (Fig. 1A, inset), with putrescine, a product of ornithine decarboxylation, and *N*-acetylornithine, a product of ornithine acetylation, being associated with, and increased by, the presence of S. gordonii (Fig. 1A). Furthermore, we noted that the presence of S. gordonii elevated the intracellular concentrations of amino acids (alanine and glutamate) and a dipeptide (alanylalanine). Additionally, 16 extracellular metabolites were found in increased concentration using a fold change cutoff of 2 and a *P* value of 0.05 (Fig. 1B). These metabolites were dominated by amino acids (ornithine, alanine, etc.) and products of amino acid fermentation and decarboxylation (butyrate, *N*-acetylputrescine, etc.). In particular, the relative concentrations of ornithine, alanine, and butyrate were markedly increased in coculture supernatants by 24.7-, 15.5-, and 9.4-fold, respectively. Further tests of S. gordonii monocultures confirmed that extracellular levels of some metabolites in coculture are not a simple addition of the metabolites in each monocultures, suggesting the presence of metabolic interactions between these two species. Specifically, S. gordonii released all amino acids described here, some of which surpassed the levels during coculture (e.g., ornithine, alanine, alanylalanine), suggesting net uptake of these metabolites by F. nucleatum (Fig. 1C). In contrast, fermented and decarboxylated products were undetected in the supernatant of S. gordonii alone, reflecting the metabolic potential of F. nucleatum to enhance production of these compounds in the presence of S. gordonii.

**FIG 2:**
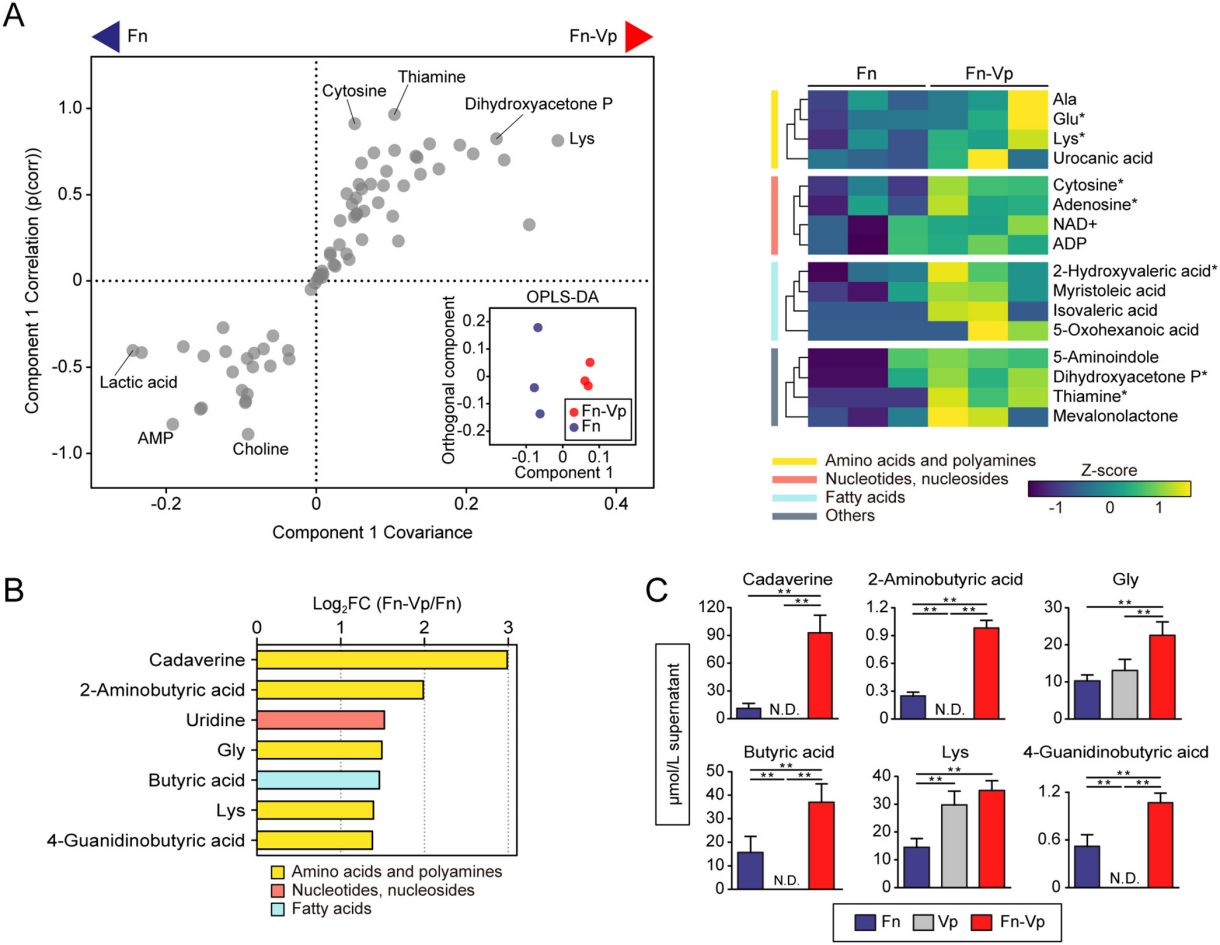
Intra- and extracellular metabolite changes of F. nucleatum cocultured with *V. parvula*. (A) Intracellular metabolite changes in F. nucleatum cocultured with *V. parvula*. OPLS-DA score plot (inset) and S-plot (left panel) show that lysine and thiamine were among the most impacted metabolites in cocultures. The right panel shows a clustered heatmap of intracellular metabolites with high reliability in the S-plot (p[corr] > 0.6). Columns represent biological replicates of F. nucleatum samples, grouped by culture method, and rows represent individual metabolites, whose levels are displayed as Z scores. *, *P* < 0.05 (Mann-Whitney U test). (B) Extracellular metabolites displaying a concentration change in cocultures compared to monocultures (log_2_ FC < −0.6, log_2_ FC > 1, and *P* < 0.05; Mann-Whitney U test). (C) Levels of the selected metabolites in spent media of cocultures and each monocultures, determined by UPLC. Error bars correspond to standard deviations. *, *P* < 0.05; **, *P* < 0.01 (one-way ANOVA with Tukey’s test). Fn, F. nucleatum subsp. *nucleatum* ATCC 25586; Vp, *V. parvula* JCM 12972.

**FIG 3:**
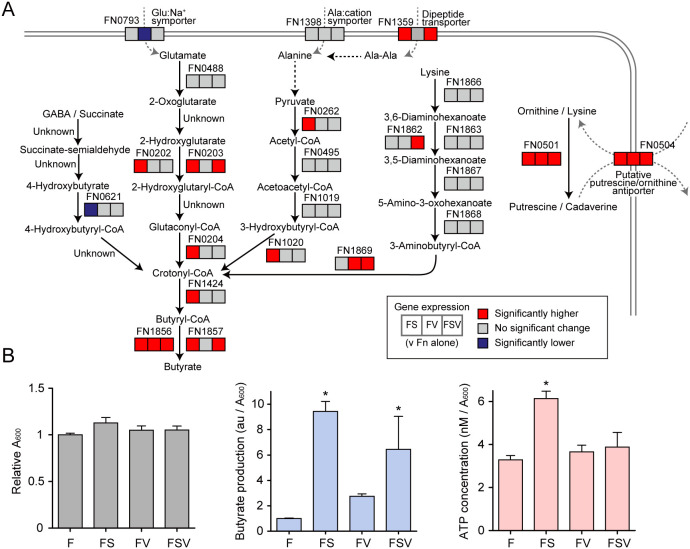
Upregulation of butyrate fermentation and polyamine production by F. nucleatum in coculture. (A) Transcriptional changes of selected genes involved in the production of butyrate and polyamines by F. nucleatum when cocultured with S. gordonii or *V. parvula* individually or in combination. Transcripts were extracted from F. nucleatum cells following the same culture conditions as those used for metabolomic assays. 16S rRNA was used for normalization. Statistical differences were analyzed using a one-way ANOVA with *post hoc* paired comparisons conducted with Dunnett’s test (*P* < 0.05). Red denotes significantly increased levels (>1.5-fold change), blue denotes decreased levels (<0.65-fold change), and gray denotes no significant changes. (B) Relative production of butyrate and ATP by F. nucleatum under each condition. The left panel shows relative absorbance changes in F. nucleatum biomass after 6 h of incubation under each condition. In this assay, biofilm cells were also retrieved to compose a total biomass. Bars are representative of three independent experiments and presented as the mean with standard deviation (SD) of three biological replicates. The center panel shows the A_600_-adjusted abundance (mean ± SD) of butyrate in culture supernatants from the metabolomics data set. The right panel shows the A_600_-adjusted ATP concentration in F. nucleatum cells after 6 h of incubation. Bars show the mean with SD of a representative experiment of five biological replicates. F, F. nucleatum alone; FS, F. nucleatum and S. gordonii; FV, F. nucleatum and *V. parvula*; FSV, F. nucleatum with S. gordonii and *V. parvula*. *, *P < *0.05 (versus F. nucleatum alone, calculated using ANOVA with Dunnett’s test).

In coculture with *V. parvula*, OPLS-DA showed a discrete intracellular metabolite profile of F. nucleatum, in which lysine, dihydroxyacetone phosphate, and thiamine were increased (Fig. 2A). Additionally, we found increased levels of seven extracellular metabolites in coculture, four of which were products of amino acid fermentation and decarboxylation. In particular, cadaverine, a product of lysine decarboxylation, exhibited the most prominent change (Fig. 2B). Since cadaverine was undetected in the supernatants of *V. parvula* monocultures (Fig. 2C), F. nucleatum is likely to produce cadaverine by utilizing lysine released by *V. parvula*. In coculture with a mixed population of S. gordonii and *V. parvula*, we observed an additive effect of these two species on the intra- and extracellular metabolic profiles of F. nucleatum (Fig. S1). Of note, any coculture showed no significant changes in F. nucleatum biomass (Fig. 3B), excluding the possibility that changes in extracellular metabolite levels were due to the increased biomass of F. nucleatum.

10.1128/msystems.00170-22.4 FIG S1 Intra- and extracellular metabolite changes of F. nucleatum cocultured with a mixture of S. gordonii and *V. parvula*. (A) Intracellular metabolite changes in F. nucleatum cocultured with mixed cultures of S. gordonii and *V. parvula*. OPLS-DA S-plot (left panel) and clustered heatmap (right panel) of intracellular metabolites with high reliability in the S-plot (p[corr] > 0.6) are shown. *, *P* < 0.05 (Mann-Whitney U test). (B) Extracellular metabolites displaying a concentration change in cocultures compared to mono-cultures (log_2_ FC < −0.6, log_2_ FC > 1, and *P* < 0.05; Mann-Whitney U test). Fn, F. nucleatum subsp. *nucleatum* ATCC 25586; Sg, S. gordonii DL1; Vp, *V. parvula* JCM 12972. Download FIG S1, TIF file, 3.0 MB. Copyright © 2022 Sakanaka et al. 2022 Sakanaka et al. https://creativecommons.org/licenses/by/4.0/ This content is distributed under the terms of the Creative Commons Attribution 4.0 International license.

### Upregulated expression of butyrate fermentation and polyamine production by F. nucleatum in coculture.

To gain further insight into these metabolic interactions, we assessed transcriptional changes in related genes of F. nucleatum using real-time reverse transcriptase PCR (RT-PCR) under the same culture conditions as those of the metabolomics assays. We selected genes for confirmation based on our metabolomic data as well as results from a previous study that used a proteomics approach to investigate S. gordonii-F. nucleatum interactions (20). In coculture with S. gordonii, we observed an upregulation of a cluster of genes encoding critical enzymes for butyrate production, including FN0202-0204, which is located in the 2-hydroxyglutarate pathway and links butyrate to glutamate (Fig. 3A), while the same trend was observed when F. nucleatum was cocultured with mixtures of S. gordonii and *V. parvula*. Since amino acid fermentation contributes to energy generation in anaerobic bacteria, we measured the ATP levels in F. nucleatum cells in coculture. We found a 1.87-fold increase in ATP levels per cell in F. nucleatum cocultured with S. gordonii (Fig. 3B). Collectively, these data indicate that coexistence with S. gordonii facilitates butyrate production, especially from glutamate, by F. nucleatum, thereby promoting ATP generation.

Putrescine and cadaverine are most commonly produced by the decarboxylation of ornithine and lysine, reactions catalyzed by ornithine decarboxylase (encoded by *speC*; Enzyme Commission number EC 4.1.1.17) and lysine decarboxylase (*cadA*; EC 4.1.1.18), respectively. F. nucleatum ATCC 25586 possesses a gene containing the domain of ornithine and lysine decarboxylases (FN0501), which shows high similarity to the sequences of both the *speC* and *cadA* genes of Escherichia coli (21, 22). The aforementioned proteomic study has also shown that FN0501 abundance was increased in a dual biofilm with S. gordonii compared with F. nucleatum alone (20). We found that the relative transcriptional level of FN0501 was elevated greater than 20-fold in all pairs of cocultures in our assays (Fig. 3A). Furthermore, a gene (FN0504) which shows high similarity to the putrescine/ornithine antiporter of E. coli (23) was also transcribed at a significantly increased level in the presence of S. gordonii and/or *V. parvula*.

Collectively, these results suggest that the presence of S. gordonii and *V. parvula* increases amino acid availability for F. nucleatum, resulting in enhanced production of fermented and decarboxylated metabolites. Notably, F. nucleatum is likely to produce putrescine and cadaverine via decarboxylation of ornithine and lysine released by S. gordonii and *V. parvula*, respectively.

### Commensal-triggered polyamine production by F. nucleatum.

**FIG 4:**
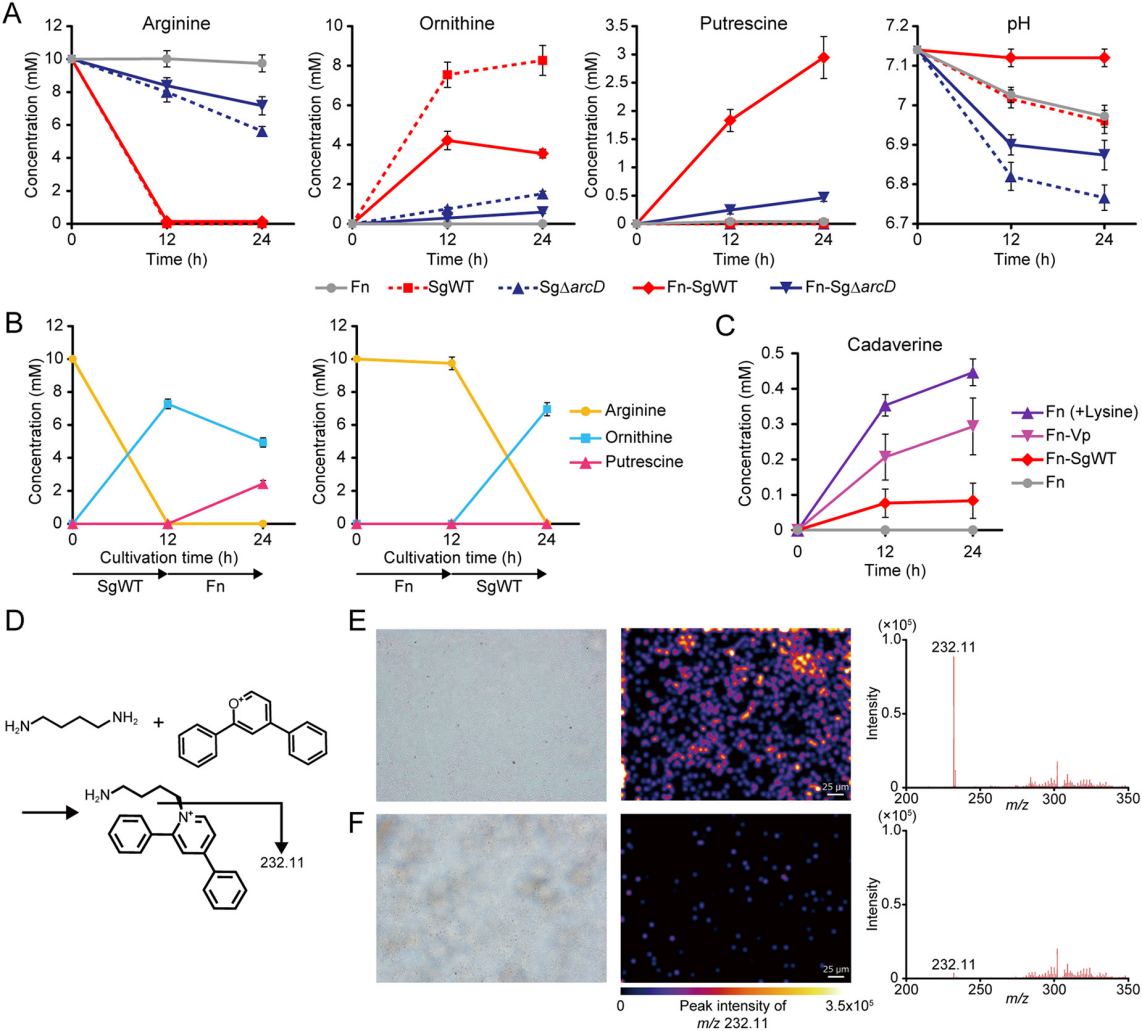
Commensal-triggered polyamine production by F. nucleatum. (A) Extracellular concentrations of arginine, ornithine, and putrescine in CDM containing 10 mM arginine incubated anaerobically for 12 and 24 h were determined by UPLC after bacterial cells were removed. Extracellular pH changes are also shown. (B) Shifts in the extracellular concentrations of arginine, ornithine, and putrescine in CDM containing 10 mM arginine incubated initially with S. gordonii or F. nucleatum for 12 h and then with its counterpart for an additional 12 h. (C) Changes in cadaverine concentrations were determined in supernatants of the designated cultures. Data are shown as the means with SDs of a representative experiment of three biological replicates. (D) Schematic of putrescine imaging. Using 2,4-diphenyl-pyranylium tetrafluoroborate (DPP-TFB), *in situ* derivatization was performed, and the distribution of putrescine (target *m/z* 232.11) was visualized through matrix-assisted laser desorption ionization mass spectrometry imaging (MALDI-MSI). (E and F) Shown are optical images and imaging results of biofilms formed on indium-tin-oxide (ITO)-coated glass slides by immersion for 24 h in F. nucleatum monocultures developed in PBS (E) with or (F) without 10 mM ornithine. Color brightness corresponds to concentration of putrescine. Fn, F. nucleatum subsp. *nucleatum* ATCC 25586; SgWT, S. gordonii DL1; SgΔ*arcD*, S. gordonii DL1 Δ*arcD* mutant; Vp, *V. parvula* JCM 12972.

We next tested whether putrescine production results from ArcD-dependent excretion of ornithine by S. gordonii. We incubated mixtures of F. nucleatum with S. gordonii wild type (WT) or Δ*arcD* as well as monocultures of each strain in CDM containing 10 mM arginine and quantified concentrations of arginine, ornithine, and putrescine in the culture supernatants. After 24 h, S. gordonii WT consumed arginine completely and released 8.26 mM ornithine but was unable to produce putrescine by itself (Fig. 4A). Similarly, F. nucleatum alone failed to utilize arginine or to produce putrescine and ornithine. In contrast, cocultures of F. nucleatum and S. gordonii WT depleted arginine and released 3.55 mM ornithine and 2.94 mM putrescine, which together with ammonia produced via ADS, allowed for maintenance of neutral pH in culture supernatants (Fig. 4A, far right). Lack of ArcD suppressed not only arginine uptake and ornithine release by S. gordonii, as demonstrated in our previous work (19), but also putrescine production in cocultures. Next, we used spent medium from monocultures of each organism to culture the other and quantified arginine, ornithine, and putrescine in the culture supernatants. S. gordonii depleted 10 mM arginine and released 7.27 mM ornithine during 12 h of cultivation (Fig. 4B). When F. nucleatum was cultured using these supernatants, ornithine decreased from 7.27 to 4.93 mM, while putrescine increased from 0 to 2.43 mM. In contrast, arginine remained intact when F. nucleatum was initially cultured, and cultivation of S. gordonii using these supernatants failed to produce putrescine. Collectively, these results indicate that production of putrescine by F. nucleatum depends on release of ornithine from S. gordonii as a metabolic by-product of the ADS.

We then incubated axenic cultures of F. nucleatum in the presence of 10 mM lysine or mixed cultures with *V. parvula* or S. gordonii WT in CDM and quantified cadaverine in the culture supernatants. After 24 h, 0.44 mM cadaverine was produced in the lysine-incubated axenic cultures, while cocultures with *V. parvula* produced 0.29 mM cadaverine, suggesting synergistic cadaverine production via lysine cross-feeding between these species (Fig. 4C).

To further validate the ability of F. nucleatum to metabolize ornithine to putrescine in biofilm microenvironments, we employed matrix-assisted laser desorption ionization mass spectrometry imaging (MALDI-MSI) and quantitatively visualized the spatial distribution of putrescine on F. nucleatum biofilms formed on glass slides treated with or without 10 mM ornithine. Illustrations of ion signals for putrescine revealed an abundance of putrescine deposited within the biofilm treated with ornithine (Fig. 4E and F), suggesting that F. nucleatum can alter the metabolic landscape in the biofilm by creating a putrescine-rich microenvironment.

### Polyamines can enhance the pathogenic potential of P. gingivalis via modulation of the biofilm phenotype.

**FIG 5:**
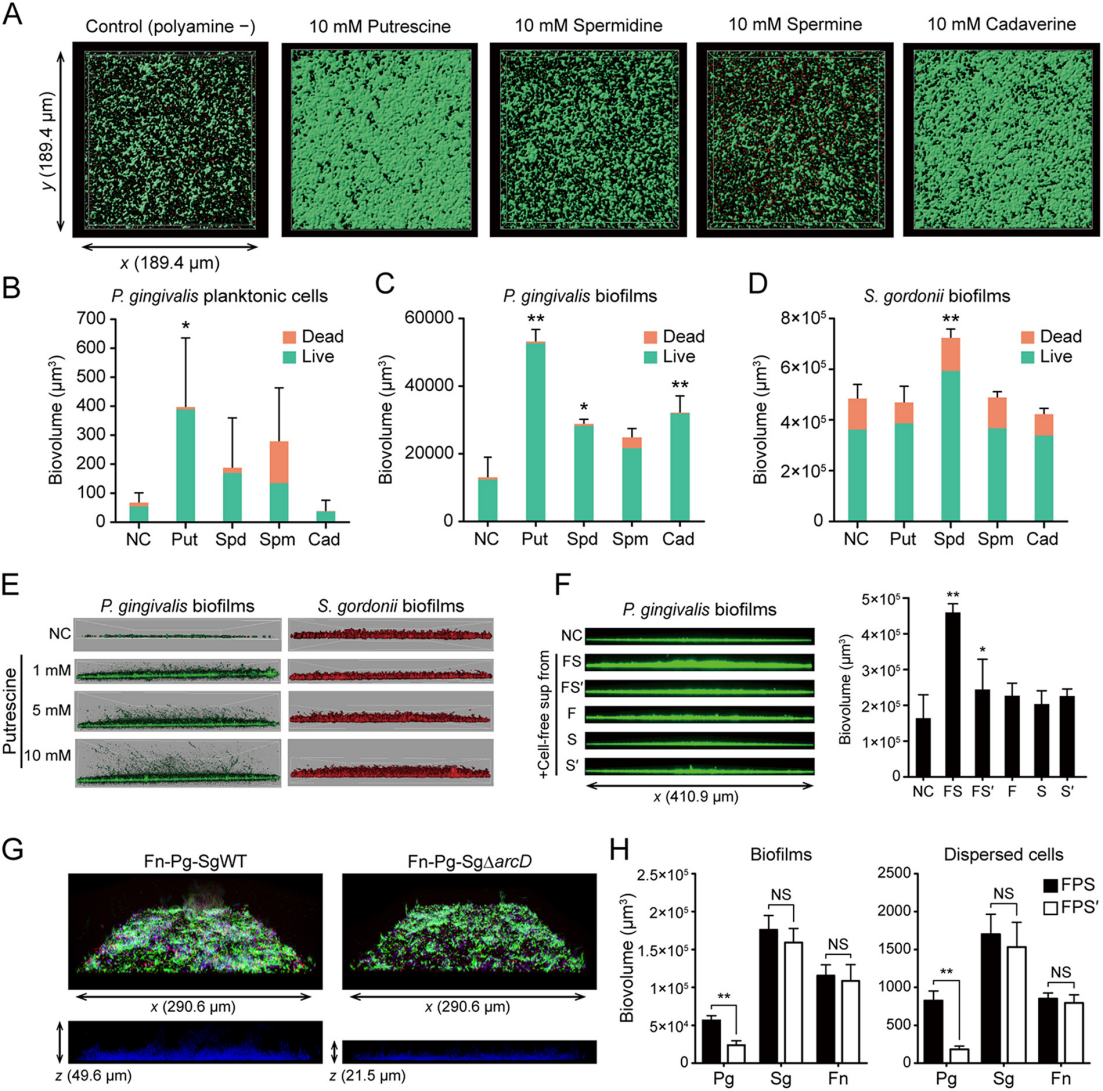
Effects of exogenous and polymicrobially produced polyamines on biofilm growth and dispersal of P. gingivalis. (A) Preformed P. gingivalis biofilms were treated anaerobically with PBS containing each polyamine for 12 h and then stained with Live/Dead dyes. A series of optical fluorescence *x*-*y* sections were collected by confocal microscopy. Images are representative of three independent experiments. (B and C) Biovolumes of dispersed planktonic cells (B) and biofilm cells (C) were measured with the Imaris Isosurface function after reconstructing three-dimensional images by applying an isosurface over Live/Dead-stained biomass separately per color (green/red). (D) The effects of each polyamine on S. gordonii biofilms were examined as a control, following the same method. Data are representative of three independent experiments and presented as the mean with SD of ten random fields from one experiment. *, *P* < 0.05; **, *P* < 0.01 compared with the control using ANOVA with Dunnett’s test. (E) Representative images of putrescine-treated biofilm microstructures of P. gingivalis and S. gordonii, which were stained with FITC and HI, respectively, at the start of the experiment. (F) P. gingivalis biofilms were formed in PBS containing 50% cell-free pH-adjusted supernatants of each culture incubated anaerobically for 24 h. FS denotes cell-free supernatants of mixed cultures of F. nucleatum and S. gordonii WT. FS′ denotes those of F. nucleatum and S. gordonii Δ*arcD*, while F, S, and S′ denote those of monocultures of F. nucleatum, S. gordonii WT and Δ*arcD*, respectively. *, *P* < 0.05, compared with the control using ANOVA with Dunnett’s test. (G) Effect of *arcD* gene deletion on tri-species biofilms of F. nucleatum, P. gingivalis, and S. gordonii. FITC-labeled F. nucleatum (green), DAPI-labeled P. gingivalis (blue) and HI-labeled S. gordonii WT/Δ*arcD* (red) were anaerobically cocultured for 48 h in biofilm medium, after which they were gently washed and further incubated for 24 h with PBS containing 10 mM arginine. Representative confocal images of tri-species biofilm architecture (above) and side-view slices of P. gingivalis localization in/above mixed biofilms (below) are shown. (H) Biovolumes of biofilms and dispersed cells of each strain quantified separately by Imaris analysis. FPS denotes tri-species biofilms of F. nucleatum, P. gingivalis, and S. gordonii WT, while FPS′ denotes those of F. nucleatum, P. gingivalis, and S. gordonii Δ*arcD*. **, *P* < 0.01 (Mann-Whitney U test).

The results presented above indicate that metabolic interactions among oral commensals can induce polyamine production by F. nucleatum. To explore the consequences of these interactions on the development of disease-associated communities, we assessed the effects of polyamines on the biofilm phenotype of a periodontal pathogen, P. gingivalis. For this experiment, we used the most widely distributed bioactive polyamines (putrescine, spermidine, spermine, and cadaverine), whose release from F. nucleatum was also confirmed in the Transwell assays (Data set S1). We incubated uniformly grown P. gingivalis biofilms anaerobically with each polyamine for 12 h in the absence of nutrition sources, in order to evaluate the action of each metabolite on P. gingivalis biofilms. After staining with Live/Dead reagent and 3 h of additional incubation with each polyamine, the amount and viability of the biofilm and of planktonic cells were evaluated using a confocal laser scanning microscope (CLSM). Analysis of the biofilm structures showed the stimulatory effects of putrescine, cadaverine (*P* < 0.01) and spermidine (*P* < 0.05) on biofilm development; in particular, exogenous putrescine caused the greatest increase in not only the viable attached biofilms but also the viable planktonic biomass, which had dispersed from the poststained biofilms (Fig. 5A and C). In contrast, cadaverine exhibited a different trend in this regard, producing more rigid biofilms with fewer suspended planktonic cells (Fig. 5B). These results suggested that these polyamines have discrete effects on the biofilm phenotype of P. gingivalis. Furthermore, using a prestained method, which excludes the possibility of the washing procedure affecting biofilm dispersal, we observed a dose-dependent effect of putrescine on biovolume as well as dispersal of cells of P. gingivalis (Fig. 5E). These results suggest a potent stimulatory effect of putrescine on biofilm life cycle, from biofilm maturation to dispersal stages. Indeed, putrescine was found to promote P. gingivalis biofilm thickening growth and dispersal even using different experimental systems, in which equally established biofilms in a 96-well polystyrene plate were treated with each metabolite for an additional 24 h (Fig. S2). To test whether the observed effects of polyamines were specific to P. gingivalis, we performed additional controls using other bacteria. Unlike P. gingivalis, S. gordonii was relatively insensitive to polyamines, and its biofilm formation was promoted only by spermidine (Fig. 5D). Meanwhile, spermine exhibited biofilm disruptive activity against F. nucleatum, and cadaverine increased both the biofilm and planktonic biomass of *V. parvula* (Fig. S3). These results indicate that polyamines have a diversity of physiological functions in different oral bacteria. Finally, we observed the response of P. gingivalis to pH-adjusted cell-free supernatants from cocultures of F. nucleatum and S. gordonii WT/Δ*arcD*. We found that cell-free supernatants from cocultures of F. nucleatum and S. gordonii WT significantly enhanced biofilm formation by P. gingivalis (Fig. 5F). Additionally, tri-species biofilms of F. nucleatum, S. gordonii, and P. gingivalis were used to determine how the loss of ArcD affects community development in the presence of arginine. Mixed biofilms were first grown by coculturing the prestained tri-species anaerobically for 48 h in an arginine-containing biofilm medium (19), after which they were further incubated with arginine as the sole nutritional source for 24 h to detect any metabolic support being provided by the other organisms in the community. Analysis of biofilm microstructures demonstrated that *arcD* deletion causes a significant reduction in P. gingivalis biomass in mixed biofilms and newly released planktonic cells, with no significant difference in other species discerned (Fig. 5G and H). Since we have previously shown that the loss of ArcD has no effect on the formation of mono-species biofilms of S. gordonii as well as dual-species biofilms with P. gingivalis (19), these data suggested that ArcD-dependent synergistic putrescine production by S. gordonii and F. nucleatum stimulates P. gingivalis biofilm development and subsequent dispersal.

10.1128/msystems.00170-22.5 FIG S2 Effects of exogenous putrescine on biofilm dispersal of P. gingivalis. Biofilms were preformed by incubating 2.8 × 10^8^ cells in 200 μL of minimal medium in a saliva-coated well of a 96-well plate for 24 h. After gentle washing with PBS, preformed biofilms were treated with prereduced PBS containing different metabolites for 24 h, followed by determination of planktonic cells in the supernatant using a spectrophotometer and biofilm biomass via crystal violet staining. **, *P* < 0.01 compared with the control using ANOVA with Dunnett’s test. NC, negative control; Put, putrescine; Cit, citrulline; Arg, arginine; Orn, ornithine. Download FIG S2, TIF file, 2.1 MB. Copyright © 2022 Sakanaka et al. 2022 Sakanaka et al. https://creativecommons.org/licenses/by/4.0/ This content is distributed under the terms of the Creative Commons Attribution 4.0 International license.

10.1128/msystems.00170-22.6 FIG S3 Effects of exogenous polyamines on biofilm growth and dispersal of F. nucleatum and *V. parvula*. (A) Preformed F. nucleatum biofilms were treated anaerobically with PBS containing each polyamine for 12 h and then stained with Live/Dead dyes. A series of optical fluorescence *x*-*y* sections were collected by confocal microscopy. Images are representative of three independent experiments. (B) Biovolumes of dispersed planktonic cells and biofilm cells were measured with the Imaris Isosurface function after reconstructing three-dimensional images. **, *P* < 0.01 compared with the control using ANOVA with Dunnett’s test. (C) Preformed *V. parvula* biofilms were treated anaerobically with PBS containing each polyamine for 12 h and then stained with Live/Dead dyes. A series of optical fluorescence *x*-*y* sections were collected by confocal microscopy. Images are representative of three independent experiments. (D) Biovolumes of dispersed planktonic cells and biofilm cells were measured with the Imaris Isosurface function after reconstructing three-dimensional images. **, *P* < 0.01 compared with the control using ANOVA with Dunnett’s test. Download FIG S3, TIF file, 3.0 MB. Copyright © 2022 Sakanaka et al. 2022 Sakanaka et al. https://creativecommons.org/licenses/by/4.0/ This content is distributed under the terms of the Creative Commons Attribution 4.0 International license.

10.1128/msystems.00170-22.7 DATA SET S1 Extracellular metabolites identified in the Transwell assay. Download Data Set S1, XLSX file, 0.03 MB. Copyright © 2022 Sakanaka et al. 2022 Sakanaka et al. https://creativecommons.org/licenses/by/4.0/ This content is distributed under the terms of the Creative Commons Attribution 4.0 International license.

### Cooccurrence of P. gingivalis with genetic modules for putrescine production by S. gordonii and F. nucleatum in plaque samples.

**FIG 6:**
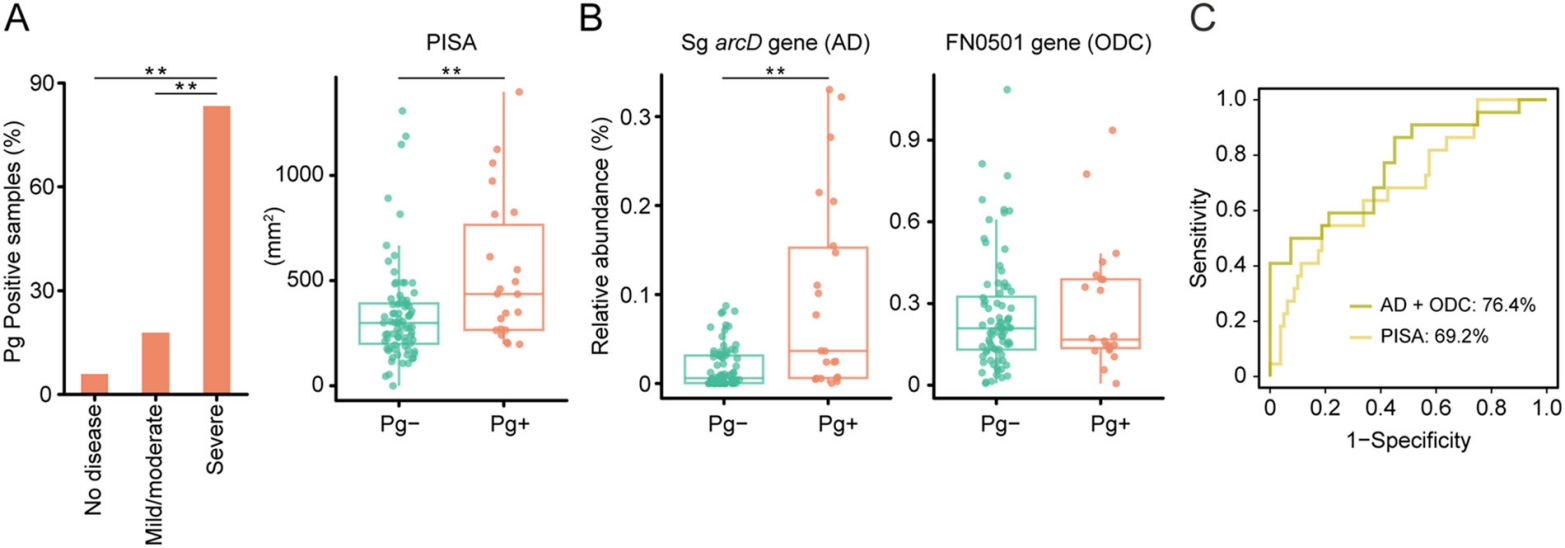
Cooccurrence of P. gingivalis with genetic modules for putrescine production by S. gordonii and F. nucleatum in 102 plaque samples. (A) Detection of P. gingivalis in supragingival biofilms in states of periodontal health, mild/moderate periodontitis, and severe periodontitis (left). Difference in periodontal inflamed surface area (PISA), a numerical representation of periodontitis severity, between P. gingivalis-positive and -negative samples (right). **, *P* < 0.01 compared with “no disease” using chi-square test (left). **, *P* < 0.01 using Mann-Whitney U test (right). (B) Difference in abundances of the S. gordonii *arcD* gene and the F. nucleatum FN0501 gene between P. gingivalis-positive and -negative samples. Real-time PCR-based-values of respective genes were divided by the total copy number of the 16S rRNA gene detected with the universal probe/primer set to obtain the relative abundance. **, *P* < 0.01 using Mann-Whitney’s U test. (C) ROC curves comparing the discriminative performance for P. gingivalis detection using logistic regression with *arcD* and FN0501 genes (olive), and PISA (yellow).

**FIG 7:**
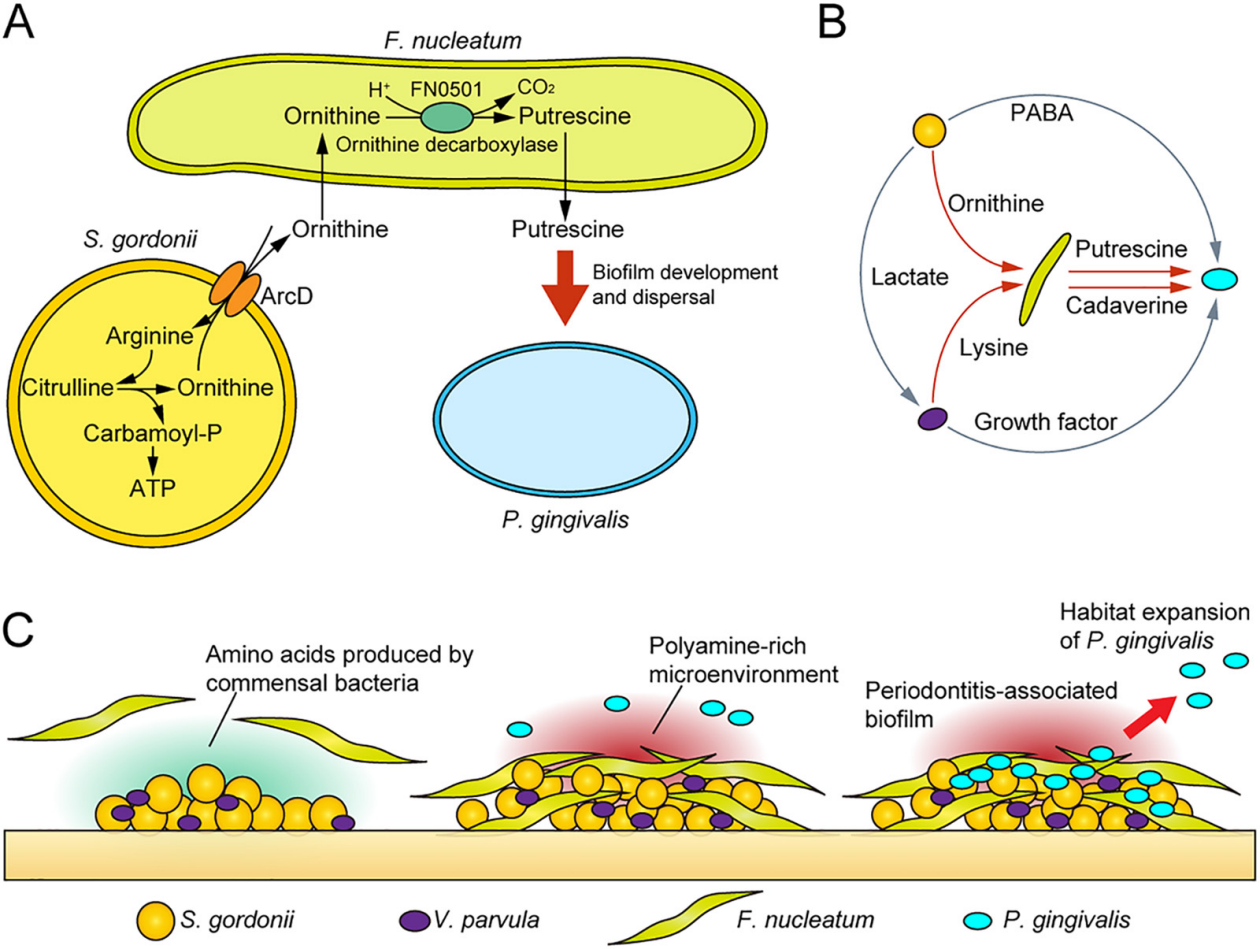
Proposed schematic model of polymicrobial metabolic synergy in the etiology of periodontal disease. (A) Pathogenic cross-feeding among three key species. The arginine deiminase system in S. gordonii facilitates putrescine production by F. nucleatum, which further promotes the biofilm overgrowth and dispersal of P. gingivalis. (B) A fuller picture of F. nucleatum-mediated trophic networks. Gray arrows denote known metabolic cross-feeding interactions, while red arrows denote those found in the present study. (C) Model depicting metabolic integration by F. nucleatum within polymicrobial communities. Commensal-triggered polyamine production by F. nucleatum contributes to shaping the periodontitis-associated community.

To test the applicability of the results to the human oral cavity, we analyzed plaque samples from 102 systemically healthy individuals using real-time PCR and investigated the relationship between the presence of P. gingivalis (detected by the P. gingivalis-specific region in the 16S rRNA gene) and the levels of the *arcD* gene of S. gordonii and the FN0501 gene of F. nucleatum relative to the total copy number of 16S rRNA in the dental plaque. We found that P. gingivalis was detected more frequently as periodontal health deteriorates (Fig. 6A). Furthermore, the *arcD* gene of S. gordonii exhibited a higher abundance in P. gingivalis-positive samples (Fig. 6B), and a combination of *arcD* and FN0501 genes by logistic regression achieved areas under the curve of 0.76 for P. gingivalis detection, surpassing the discriminative performance of periodontal inflamed surface area (PISA), a numerical measure of periodontal inflammation (24), as well as a possible indicator of dysbiotic microbiota (3) (Fig. 6C). These data provide clinical evidence suggesting cooccurrence of P. gingivalis with genetic modules for putrescine production by S. gordonii and F. nucleatum. Based on these results, we propose a model of metabolic interactions within oral biofilms whereby ADS in S. gordonii facilitates putrescine production by F. nucleatum, which could further promote the biofilm overgrowth and dispersal of P. gingivalis (Fig. 7A and B).

## DISCUSSION

Amino acids are the main source of energy for F. nucleatum, but this species does not possess a high level of endopeptidase activity (25), and previous studies propose that it takes advantage of amino acids and peptides available through interspecies interactions with proteolytic bacteria such as P. gingivalis (26, 27). Combining the results of intra- and extracellular metabolite changes in F. nucleatum from Transwell assays suggested possible engagement of some amino acids in cross-feeding interactions. Specifically, in addition to ornithine, consistent with our previous report (19), F. nucleatum acquired alanine, alanylalanine, and glutamate released from S. gordonii and lysine from *V. parvula*, suggesting that amino acids can also be supplied by oral commensals lacking proteolytic activity through a cross-feeding behavior. Indeed, emerging evidence suggests that amino acid cross-feeding is one of the main drivers of interspecies interactions in microbial communities (5, 28, 29). Recent computational modeling work shows that resource-poor environments can provide the basis for the release of a wide variety of metabolites, including amino acids, without a fitness cost, by diverse microbial species. Release of these metabolites generates ample cross-feeding opportunities, which can be facilitated by anoxic conditions (30). In light of this, our results support the plausibility of widespread amino acid cross-feeding within both the supragingival and subgingival communities, which could underlie metabolic shifts during the transition from periodontal health to disease.

One of the most striking findings in this study was that S. gordonii and F. nucleatum interact cooperatively to produce putrescine from arginine through ornithine, and this trophic web results in alterations in P. gingivalis biofilm phenotypes. Conversion of ornithine to putrescine via decarboxylation consumes cytoplasmic protons and creates a proton motive force (31), offering an acid resistance and an energetic advantage to F. nucleatum. Consumption of ornithine also helps maintain the ADS function and achieve a sustainable energy supply for S. gordonii. From ecological and evolutionary perspectives, therefore, this collaborative metabolism accomplished by the ADS of S. gordonii and ornithine decarboxylase of F. nucleatum would be favored by natural selection, since it allows for the efficient use of limited resources and confers fitness benefits to both species. Notably, we confirm that many clinically important subspecies, including F. nucleatum subsp. *vincentii*, *animalis*, and *polymorphum*, possess gene sequences exhibiting over 98% identity with FN0501. Additionally, a recent analysis has shown that F. nucleatum subsp. *polymorphum* has high ornithine decarboxylase activity with the FN0501 homologue protein (32). Hence, other subspecies of F. nucleatum are highly likely to have the ability to convert ornithine to putrescine. Moreover, a number of oral bacteria are ADS positive, including Streptococcus sanguinis, Streptococcus mitis, and several *Lactobacillus*, *Actinomyces*, and *Saccharibacteria* (TM7) species (33, 34), indicative of the potential engagement of species other than S. gordonii in synergistic putrescine production. Interestingly, recent work provides experimental evidence showing that acquisition of the ADS by TM7 likely enables the transition from environmental origin to the oral cavity, underscoring the importance of the ADS over the course of microbiome assembly in the oral environment (35). The findings of the present study indicate that the ADS-driven sequential reaction eventually creates a microenvironment favoring the survival of P. gingivalis with consequences for periodontal health, offering further evidence that arginine catabolism can impact microbiome assembly and host-microbiome interactions in the oral environment.

It should be noted that significant extracellular accumulation of putrescine was observed in polyamine production assays (Fig. 4) while not in Transwell assays (Fig. 1B). Given that polyamines have diverse cellular functions and their intracellular levels are strictly regulated in bacteria (36), one possible explanation is that putrescine secretion by F. nucleatum is likely due to metabolic overflow, where a certain amount of ornithine in the medium triggers overproduction of putrescine, inducing its secretion (37). We consider that the extracellular level of ornithine had yet to reach this amount in Transwell assays, where F. nucleatum and S. gordonii were incubated without arginine for only 6 h, though additional studies are required to elucidate the underlying mechanisms regulating production and secretion of putrescine in this organism.

Putrescine is regarded as one of the most common polyamines in bacteria, and together with spermidine, its biosynthesis was found to be essential for the growth of many bacteria (38, 39). Putrescine and spermidine are also required for biofilm formation by Bacillus subtilis and Yersinia pestis (40–42), although spermidine inhibits biofilm formation by some bacteria (43, 44). Here, we demonstrated that exogenous putrescine and cadaverine stimulate P. gingivalis biofilm development while producing different biofilm phenotypes; cadaverine yielded more rigid biofilms with fewer suspended cells, whereas putrescine thickened biofilms with more suspended cells. The distinct biofilm phenotypes may represent differences in biofilm developmental stages and suggest the potential of putrescine to accelerate the biofilm life cycle of maturation and dispersal. In fact, previous work showed that putrescine acts as an extracellular signal for swarming and is necessary for effective migration across agar surfaces in Proteus mirabilis (45). A recent multiomics study showed that P. gingivalis strain 381 can surface translocate when sandwiched between two surfaces, and this dispersion-like behavior involves intracellular metabolic changes in the arginine and polyamine pathways, with citrulline and ornithine accumulation along with exhaustion of arginine and putrescine (46). Although the mechanistic details of the role of polyamines in P. gingivalis physiology are largely unknown and further studies will be necessary to gain a better understanding, putrescine seems to be a key signal for transforming physiology and accelerating the biofilm life cycle of P. gingivalis to promote habitat expansion.

A number of studies using clinical samples have found the possible involvement of polyamines and related metabolites in the pathogenesis of periodontitis. A comparative metagenomics study revealed that a periodontitis-associated microbiota exhibits distinct metabolic functions that include polyamine uptake systems regulated by a putrescine transport ATP-binding protein (47). A metabolomic analysis of gingival crevicular fluids revealed significantly elevated levels of putrescine and cadaverine, as well as various amino acids, including ornithine, in the subgingival crevice of periodontitis sites (48). Our previous metabolomic studies using saliva samples also showed that a disease-associated microbiota likely produces polyamines, including putrescine and cadaverine, which is reflected in the distinct salivary metabolomic landscapes of periodontitis patients (49, 50). Although the extent to which F. nucleatum dictates the enrichment of polyamine metabolism in periodontitis has yet to be determined and other community members may contribute to polyamine production, the data presented in this work add to the evidence that the transition from periodontal health to disease is linked to metabolic specialization, including polyamine metabolism, in subgingival microbial communities.

Although this study focused on a few oral bacteria to simulate metabolic cross-feeding during dental biofilm maturation, we acknowledge that oral biofilm ecosystems have food webs comprising many layers of complexity that fall outside the scope of our framework (51). In addition, the nature of metabolic interactions may be affected by the physical proximity of species and their structural organization, which are important features of biofilms (52). Furthermore, while we used glucose as the carbon source in CDM, we should consider using different carbon sources, given the diversity of nutrient sources found in the oral cavity, as well as the reported saccharolytic capacity of F. nucleatum against fructose (53). These limitations notwithstanding, this study provides new insights into how the trophic web in oral biofilm ecosystems impacts the process of dental biofilm maturation; specifically, ornithine cross-feeding by S. gordonii induces putrescine production by F. nucleatum, which can culminate in the overgrowth and habitat expansion of P. gingivalis. Our results reveal a new example of cooperative metabolism between oral bacteria that is unattainable without the sharing of metabolic pathways in multiple taxa and shed light on the metabolic aspects of F. nucleatum in the context of the pathogenicity of microbial communities through metabolic communications within oral biofilms.

## MATERIALS AND METHODS

### Bacterial strains and growth conditions.

F. nucleatum subsp. *nucleatum* ATCC 25586, P. gingivalis ATCC 33277, *V. parvula* JCM 12972, and S. gordonii DL1 and its isogenic Δ*arcD* mutant (19) were used in this study. F. nucleatum, P. gingivalis, and *V. parvula* were cultivated on anaerobic CDC 5% sheep blood agar (Becton, Dickinson and Company, Franklin Lakes, NJ, USA) at 37°C in an anaerobic chamber (Concept Plus; Ruskinn Technology, Bridgend, UK) with an atmosphere containing 10% H_2_, 10% CO_2_, and 80% N_2_. Liquid cultures of F. nucleatum were anaerobically grown at 37°C in brain heart infusion broth (Becton, Dickinson and Company) supplemented with yeast extract, Trypticase peptone, Biosate peptone (10 g/L each), basal solution (72 μM CaCl_2_, 66 μM MgSO_4_, 0.23 mM K_2_HPO_4_, 0.29 mM KH_2_PO_4_, 4.76 mM NaHCO_3_, 1.37 mM NaCl), hemin (5 mg/L), and menadione (1 mg/L). Liquid cultures of *V. parvula* and P. gingivalis were anaerobically grown at 37°C in DSMZ medium 136, and Trypticase soy broth supplemented with yeast extract (1 g/L), hemin (5 mg/L), and menadione (1 mg/L), respectively. S. gordonii strains were grown statically in liquid or on agar-solidified Todd-Hewitt broth under aerobic conditions at 37°C, and erythromycin (5 mg/L) was used for selection. At the early-stationary phase, the cells were harvested by centrifugation, washed twice with prereduced phosphate-buffered saline (PBS), and then used in the assays. For the Transwell assays, bacteria were anaerobically cultured at 37°C in CDM [0.1% glucose, 58 mM K_2_HPO_4_, 15 mM KH_2_PO_4_, 10 mM (NH_4_)_2_SO_4_, 35 mM NaCl, 0.1 mM MnCl_2_ · 4H_2_O, 2 mM MgSO_4_ · 7H_2_O, 40 μM nicotinic acid, 0.1 mM pyridoxine-HCl, 10 μM pantothenic acid, 1 μM riboflavin, 0.3 μM thiamine-HCl, 0.05 μM d-biotin]. All the media and PBS used for culture and washing of anaerobes in this study were prereduced in an anaerobic chamber.

### Metabolomic and transcriptional analyses in Transwell assay.

Synthetic communities were created by inoculating 1.4 × 10^10^ cells of F. nucleatum in 2.6 mL of CDM per well in the lower chamber of a 6-well Transwell system with 0.4-μm-pore polystyrene membrane inserts (Corning, New York, USA), into which 1.4 × 10^10^ cells of S. gordonii or *V. parvula* individually or their mixture (7 × 10^9^ cells each) in 1.5 mL of CDM per well or an equal volume of fresh CDM (as a control) were added. Conditions were in triplicate, and the setup was anaerobically incubated at 37°C. Anaerobic conditions were intended not only to reproduce ornithine cross-feeding (19), but to protect F. nucleatum from toxicity of H_2_O_2_ produced by S. gordonii in the presence of oxygen (54), which is unlikely to occur in the anaerobic microenvironment of the gingival margins and subgingival area, and to maximize the cooperative potential for metabolite exchange between these species. After 6 h, F. nucleatum cells were collected by pipetting from the lower chamber and washed with Milli-Q water by centrifugation. For metabolomics analysis, bacterial pellets were immediately fixed by adding methanol containing 5 μM internal standard. Spent medium from cultures and sterile CDM were centrifuged, filtered through 0.22-μm polyethersulfone (PES) filtration devices (Merck Millipore, Darmstadt, Germany), and lyophilized. Capillary electrophoresis time-of-flight mass spectrometry (CE-TOFMS) was performed using an Agilent CE-TOFMS system equipped with a fused silica capillary (50 μm [inside diameter (i.d.)] × 80 cm] as previously described (19). The conditions for the measurement of cationic/anionic metabolites were as follows: running buffer, a solution composed of cation buffer solution (H3301-1001; Human Metabolome Technologies [HMT; Tsuruoka, Japan]) and anion buffer solution (H3302-1023); CE voltage, +27 kV/+30 kV; MS ionization, electrospray ionization (ESI) positive/ESI negative; MS capillary voltage, 4,000 V/3,500 V; MS scan range, *m/z* 50 to 1,000; and sheath liquid, HMT sheath liquid (H3301-1020). Identification of metabolites and evaluation of the relative amounts were conducted using Master Hands (versions 2.16.0.15 and 2.17.1.11; Keio University, Tokyo, Japan) with the HMT metabolite database. The relative amount of each metabolite was calculated with reference to the internal standard material (HMT). As for metabolites whose levels were altered significantly in spent media of cocultures, replicate experiments were performed with the additional control of monocultures of S. gordonii or *V. parvula* in CDM (1.4 × 10^10^ cells in the lower chamber of a Transwell plate), and metabolite concentrations in the culture supernatants were quantified using an Acquity ultraperformance liquid chromatography (UPLC) system with a photodiode-array (PDA) detector (Waters, Milford, MA, USA), as described previously (19), with the exception of butyrate, which was quantified using high-performance liquid chromatography as described previously (55).

Quantification of mRNA transcripts was performed by real-time RT-PCR as described previously (19). Briefly, strains were cultured and harvested in triplicate under the same conditions as those for the metabolomics analysis. F. nucleatum cells were immediately frozen in liquid nitrogen and homogenized with zirconia beads at 1,500 rpm for 5 min at 4°C using a Shake Master Neo device (BioMedical Science, Tokyo, Japan). Total RNA was isolated and converted to cDNA with an iScript cDNA synthesis kit (Bio-Rad, California, USA). Real-time RT-PCR was performed on a Roter-Gene Q system (Qiagen, Hilden, Germany) by the ΔΔ*CT* method using 16S rRNA as an internal control. Primers are listed in Table S1. ATP was measured in a chemiluminescent assay as described previously (56). Briefly, cocultures were incubated for 6 h by the method described above, and a 20-μL aliquot of F. nucleatum cultures was harvested and mixed with 180 μL of dimethyl sulfoxide. ATP was measured by mixing samples 1:1 with BacTiter-Glo reagent (Promega, Madison, WI, USA) in a 96-well opaque white microtiter plate. Luminescence was measured using a plate reader (Wallac 1420 ARVO; PerkinElmer, Waltham, MA, USA). A standard curve was generated with each plate measurement using a known concentration of ATP.

10.1128/msystems.00170-22.1 TABLE S1 Primers used for real-time RT-PCR in the Transwell assays. Download Table S1, DOCX file, 0.03 MB. Copyright © 2022 Sakanaka et al. 2022 Sakanaka et al. https://creativecommons.org/licenses/by/4.0/ This content is distributed under the terms of the Creative Commons Attribution 4.0 International license.

### Polyamine production assay.

Monocultures of F. nucleatum or S. gordonii (6.75 × 10^9^ cells) and mixed cultures of F. nucleatum with S. gordonii or *V. parvula* (6.75 × 10^9^ cells each) were anaerobically incubated at 37°C in prereduced CDM containing 10 mM arginine or lysine. Culture supernatants were collected by centrifugation and filter-sterilized with 0.22-μm PES filters (Merck Millipore). Metabolite concentrations in the culture supernatants were determined using an Acquity UPLC system with a PDA detector (Waters), as described previously (19). pH values in the culture supernatants were determined with an F51 pH meter (Horiba, Kyoto, Japan).

### MALDI-MSI for putrescine visualization.

MALDI-MSI was performed as described previously (57). Briefly, indium-tin-oxide (ITO)-coated glass slides were immersed in F. nucleatum monocultures (2 × 10^9^ cells/mL) incubated anaerobically in prereduced PBS with or without 10 mM ornithine. After 24 h, biofilms formed on the glass slides were gently washed with PBS and subjected to fixation. After preparation of the sample, on-biofilm derivatization was performed. To derivatize amine groups, 2,4-diphenyl-pyranylium tetrafluoroborate (DPP-TFB; Merck Millipore) was used. DPP-TFB was dissolved in methanol to prepare 10-mg/mL stock solutions. DPP-TFB solutions used for derivatization contained 6 μL of the stock solution, 69 μL of 60% methanol, and 1 μL of triethylamine. *In situ* derivatization was performed with 50 μL of the DPP-TFB solution deposited manually onto each biofilm using an airbrush (PS-270; GSI Creos, Tokyo, Japan). The sample slides were then incubated at room temperature (25°C) for 60 min. After incubation, α-cyano-4-hydroxycinnamic acid (CHCA; Merck Millipore) as a matrix was sublimated using an iMLayer system (Shimadzu, Kyoto, Japan) for MALDI. The matrix layer was 0.5 μm on the biofilm surface. Following sublimation, 50 μL of matrix solution (10 mg/mL CHCA in a solution containing 30% acetonitrile, 10% 2-propanol, and 0.1% formic acid) was sprayed onto each biofilm with the airbrush. MALDI-MSI analyses were performed using an iMScope TRIO (Shimadzu). This instrument has a sample chamber to observe with a microscope and a MALDI ionization source with an Nd:YAG laser (λ = 355 nm and 1 kHz) under atmospheric pressure. The biofilm surface was irradiated with the laser, with 80 shots for each data point. Mass spectra for DPP derivatization were acquired in the positive ion detection mode in the mass range of *m/z* 100 to 350. In the MSI experiments, constant voltages for the sample stage and detector were applied at 3.50 kV and 2.1 kV, respectively. The laser power was kept at a constant of 25 (arbitrary unit in iMScope TRIO). To obtain a DPP-putrescine signal, MS/MS analysis was performed using selected precursor ions of DPP-putrescine ([M]+ 303.19), and the fragment ion was detected at *m/z* 232.11. After the experiment, peak intensity maps were reconstructed using IMAGEREVEAL (Shimadzu).

### Biofilm assay.

To assess the effects of various polyamines on P. gingivalis biofilms, we initially preformed biofilms by incubating 4 × 10^7^ cells anaerobically at 37°C for 24 h in prereduced dedicated minimal medium (58) in a 25% saliva-coated well of an 8-well chamber slide system (59) (Thermo Fisher Scientific, Waltham, MA, USA) with rotating. The medium was gently replaced with prereduced PBS containing each polyamine, and biofilms were further incubated anaerobically for 12 h. After staining with a Live/Dead BacLight kit (Molecular Probes, Eugene, OR, USA) and gentle washing with PBS and extended anaerobic incubation in prereduced PBS containing each polyamine for 3 h, biofilm microstructures and newly released planktonic cells were imaged at 10 different randomly selected regions per each group using a Leica SP8 CLSM (Leica Microsystem, Wetzlar, Germany) and analyzed with Imaris version 7.1.0 software (Bitplane, Belfast, UK). An isosurface was applied over Live/Dead-stained biomass separately per color (green/red). The conditions for measurement of planktonic-cell biovolume were as follows. A 190-μm by 190 μm by 7 μm space (in x by *y* by *z*) above the biofilm surface was set as an area of interest, where isosurface particles of the approximate single size of an individual P. gingivalis cell (0.7 μm^3^ to 4.0 μm^3^) were quantified. For measurement of biofilm-cell biovolume, isosurface particles larger than the quintuple size of P. gingivalis planktonic cells (over 5.0 μm^3^) were quantified within the biofilm layer. For other bacteria, the same procedures were repeated, except the following: for biofilm preformation, S. gordonii (4 × 10^7^ cells) was incubated in prereduced dedicated minimal medium (19). As for F. nucleatum and *V. parvula*, 2 × 10^8^ cells were incubated anaerobically at 37°C for 24 h in prereduced PBS.

To assess the effects of putrescine on biofilms, S. gordonii and P. gingivalis were stained with 15 mg/L hexidium iodide (HI; Thermo Fisher Scientific) and 4 mg/L 5- (and 6)-carboxyfluorescein and succinimidyl ester (FITC; Thermo Fisher Scientific), respectively. Preformed biofilms were treated with prereduced PBS containing putrescine for 12 h, followed by CLSM. To validate the effects of putrescine on biofilm dispersal of P. gingivalis, biofilms were preformed by incubating 2.8 × 10^8^ cells in 200 μL of minimal medium (58) in a saliva-coated well of a 96-well plate for 24 h. After gentle washing with PBS, preformed biofilms were treated with prereduced PBS containing different metabolites for 24 h, followed by determination of planktonic cells in the supernatant using a spectrophotometer (UV-1600; Shimadzu), and biofilm biomass via crystal violet staining using a plate reader (Wallac 1420 ARVO; PerkinElmer). To observe the responses of P. gingivalis to cell-free supernatants from 24-h cultures of F. nucleatum and S. gordonii, the culture supernatants were obtained by the same method as those of the polyamine production assays, with the pH adjusted to 7. P. gingivalis (2.8 × 10^8^ cells) was stained with FITC and incubated anaerobically for 24 h in prereduced PBS containing 50% cell-free supernatants, followed by CLSM. For analysis of mixed biofilm formation, FITC-labeled F. nucleatum (10^7^ cells), DAPI (4′,6-diamidino-2-phenylindole)-labeled P. gingivalis (10^7^ cells), and HI-labeled S. gordonii (4 × 10^7^ cells) were anaerobically cocultured for 48 h in arginine-containing CDM dedicated for mixed biofilm experiments (19). After gentle washing and further anaerobic incubation for 24 h with prereduced PBS containing 10 mM arginine, biofilm microstructures and dispersed cells were visualized using CLSM and analyzed with Imaris software (Bitplane).

### Study participants and detection of selected genes.

We employed supragingival plaque samples, collected in our previous multiomics study (49, 50), which was conducted from 2013 to 2014, with approval from the Osaka University Research Ethics Committee and in accordance with the principles of the Helsinki Declaration and STROBE guidelines for human observational studies. All participants provided written informed consent prior to enrollment and provided samples at Osaka University Dental Hospital. Participants were recruited from volunteers employed by Osaka University or individuals visiting the clinic of Preventive Dentistry at Osaka University Dental Hospital. Exclusion criteria included abnormal salivary function, use of antibiotics within the previous 3 months, use of prescription drugs within the previous 2 weeks, past and/or present use of cigarettes, diagnosis of any disease in oral soft tissues, or the presence of other systemic conditions. Five calibrated and licensed dentists performed oral examinations, including full-mouth general dental survey and detailed periodontal assessment, as well as plaque collection. All subjects were asked to refrain from eating, drinking, or brushing for at least 1 h prior to undergoing these procedures. Periodontal assessment included recording of probing depths, bleeding on probing, gingival recession, and clinical attachment level at 6 sites of all teeth present. Those results were used for calculating PISA (24) and defining cases of periodontitis (60). Supragingival plaque samples along the gingival margin were collected from throughout the mouth after oral examinations using a Gracey curette (11/12 Mini Five; Hu-Friedy, Chicago, IL, USA). All samples were frozen with liquid nitrogen and stored at −80°C until analysis. The final study population consisted of 102 individuals from an initial sample of 109, as 7 had either missing clinical parameter values or plaque samples (Table S2). Bacterial DNA was extracted using the DNeasy PowerSoil Pro kit (Qiagen) protocol according to the manufacturer’s instructions. Primers and TaqMan probes (conjugated with FAM, ZEN, and IBFQ) were designed based on the specific sequences for *arcD* of S. gordonii, FN0501 of F. nucleatum, and the 16S rRNA gene of P. gingivalis using nucleotide BLAST (NCBI), ClustalW (DDBJ), and PrimerQuest (Integrated DNA Technologies, Coralville, IA, USA). A previously described universal probe/primer set was designed with some modifications and used for standardization (61, 62). TaqMan real-time PCR was performed on a Roter-Gene Q system (Qiagen) using a Thunderbird SYBR qPCR Mix (Toyobo, Osaka, Japan). Primers and probes are listed in Table S3. The resulting quantitative values of *arcD* and FN0501 genes were divided by the total copy number of the 16S rRNA gene detected with the universal probe/primer set to obtain the relative abundance in plaque samples, which was subsequently used for logistic regression and receiver operating characteristic (ROC) curves. For the detection of P. gingivalis, a resulting value of zero was considered negative, and a value greater than zero was considered positive. In ROC analysis, logistic regression was performed to make a quantitative summary of the relative levels of *arcD* and FN0501 genes.

10.1128/msystems.00170-22.2 TABLE S2 Characteristics of the study participants. Download Table S2, DOCX file, 0.03 MB. Copyright © 2022 Sakanaka et al. 2022 Sakanaka et al. https://creativecommons.org/licenses/by/4.0/ This content is distributed under the terms of the Creative Commons Attribution 4.0 International license.

10.1128/msystems.00170-22.3 TABLE S3 Primers and probes used for analysis of plaque samples. Download Table S3, DOCX file, 0.03 MB. Copyright © 2022 Sakanaka et al. 2022 Sakanaka et al. https://creativecommons.org/licenses/by/4.0/ This content is distributed under the terms of the Creative Commons Attribution 4.0 International license.

### Statistical analyses.

Statistical analysis for intracellular metabolomics data was based on multivariate analysis by OPLS-DA using SIMCA-P software (version 14.0; Umetrics, Umeå, Sweden). Score plots and S-plots were constructed using Pareto scaling, and metabolites that contributed most to discrimination were chosen based on a loading value scaled as a correlation coefficient between each metabolite and the predictive component of the model [p(corr) >0.6]. Statistical analysis for extracellular metabolomics data was based on comparison between groups with Mann-Whitney U test using SPSS (version 22; IBM, New York, USA). Extracellular levels of selected metabolites were compared between cocultures and each monoculture with one-way analysis of variance (ANOVA) followed by Tukey’s test using SPSS. The results from real-time RT-PCR and biofilm assays were analyzed by one-way ANOVA with *post hoc* paired comparison conducted with Dunnett’s test using SPSS. ROC curves and logistic regression were performed with the R package (version 4.0.3).

### Data availability.

Metabolomics data are available at Metabolomics Workbench (study ID ST001260 and ST001261). A STORMS (Strengthening the Organizing and Reporting of Microbiome Studies) checklist is available at https://zenodo.org/record/6602629#.Ysl0wXZBxHY.

10.1128/msystems.00170-22.8 DATA SET S2 Intracellular metabolites of F. nucleatum identified in the Transwell assay. Download Data Set S2, XLSX file, 0.02 MB. Copyright © 2022 Sakanaka et al. 2022 Sakanaka et al. https://creativecommons.org/licenses/by/4.0/ This content is distributed under the terms of the Creative Commons Attribution 4.0 International license.
