# Supplementary figures and images for: Fusobacterium nucleatum Metabolically Integrates Commensals and Pathogens in Oral Biofilms
Source: mSystems. 2022 Jul 19;7(4):e00170-22. doi: 10.1128/msystems.00170-22 (PMC9426547; doi:10.1128/msystems.00170-22)

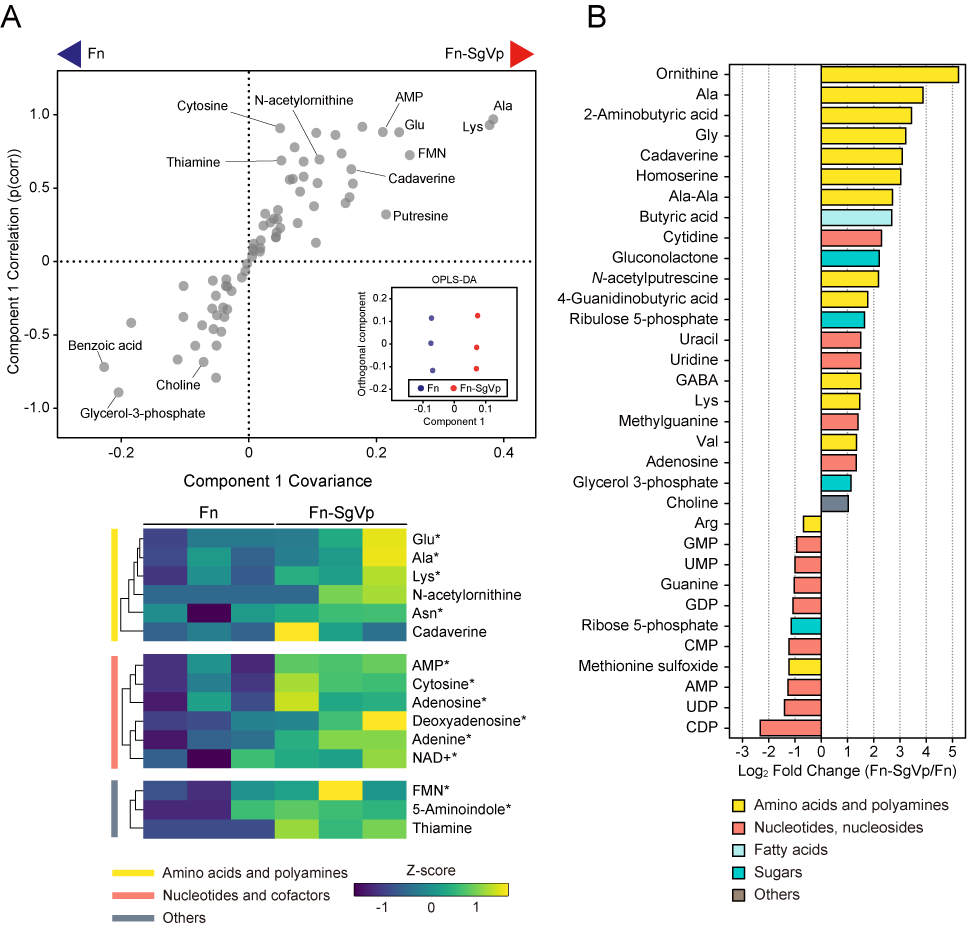

Supplement: FIG S1 [file msystems.00170-22-s0001.tif]

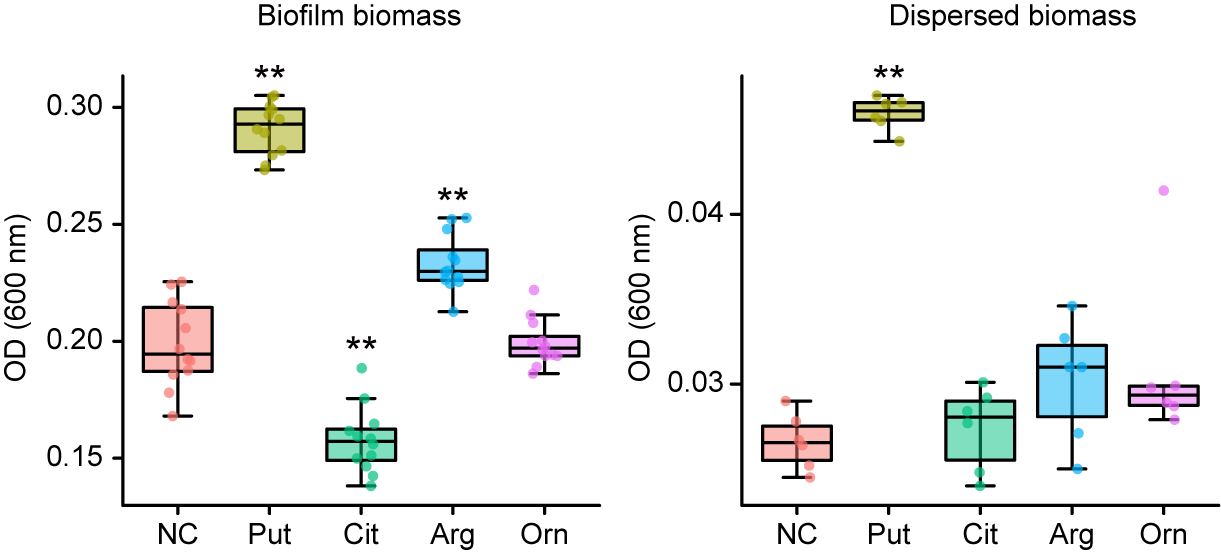

Supplement: FIG S2 [file msystems.00170-22-s0002.tif]

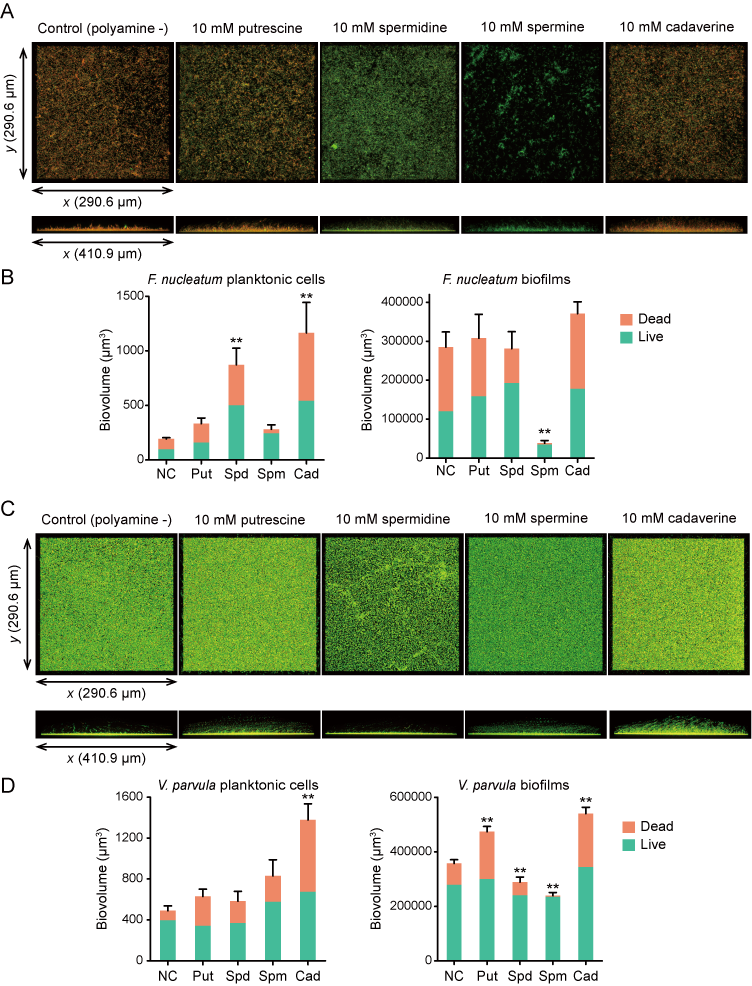

Supplement: FIG S3 [file msystems.00170-22-s0003.tif]
