# Supplementary material for: Fusobacterium nucleatum Metabolically Integrates Commensals and Pathogens in Oral Biofilms
Source: mSystems. 2022 Jul 19;7(4):e00170-22. doi: 10.1128/msystems.00170-22 (PMC9426547; doi:10.1128/msystems.00170-22)
Supplement: TABLE S1 [file msystems.00170-22-s0004.docx]

**Table S1.** Primers used for real-time RT-PCR in the Transwell assays

| ORF | Direction | Sequence (5′-3′) | Product size (bp) | Source |
| --- | --- | --- | --- | --- |
| FN0202 | F | AAGCCTTATGCGGCTGTTTA | 88 | This study |
|  | R | TATCCCAATCTCCACCTGCT |  |  |
| FN0203 | F | GCTGTTCAATGGCCAAATGT | 95 | This study |
|  | R | TGAGCTCCTCCAATGAAAGC |  |  |
| FN0204 | F | GAGCAGGAATTGTTGGAGGA | 89 | This study |
|  | R | TTTAGCGGCCATTGTAGCTT |  |  |
| FN0262 | F | GGAAGACCTGCTGAAACTGC | 86 | This study |
|  | R | GCAGCACCATTTTGGTCTTT |  |  |
| FN0488 | F | TATGGCTTGGATGCAAGATG | 122 | This study |
|  | R | CACCAAATCCAGTTGCTTCA |  |  |
| FN0495 | F | ATGAAGCATTTGCTGCTCAA | 133 | This study |
|  | R | GCTCCAACTGGGTGTCCTAA |  |  |
| FN0501 | F | ATGGGAGAGGCTCCTTTTTC | 116 | This study |
|  | R | ACCCCATAAGCATCTGCAAC |  |  |
| FN0504 | F | GCAGTAACTTTTGCAGCTGCT | 147 | This study |
|  | R | TGCCATAACTCCCCAAGCAA |  |  |
| FN0621 | F | GGTGGGCAAGTTGACTTTGT | 81 | This study |
|  | R | TCCCGTAGTTGATGGGAGAG |  |  |
| FN0793 | F | GGTGGAGTTGGAGTTGCATT | 170 | This study |
|  | R | GGTCCAAATGCTCCTGATGT |  |  |
| FN1019 | F | GCATTTGCACAAACTGAAGG | 139 | This study |
|  | R | TGTCAGCATCTGCTTGTTCC |  |  |
| FN1020 | F | TTGCTTTAGGTGGAGGCTGT | 119 | This study |
|  | R | AGTTCCACCAAATCCAGGTG |  |  |
| FN1359 | F | TGGAATGCACCAGAAGAACA | 196 | This study |
|  | R | AAGTCGCCCACTCAATCATC |  |  |
| FN1398 | F | AGGACAGGTTTTGCACAAGG | 150 | This study |
|  | R | TCCAACGTGAGAAGCAACAG |  |  |
| FN1424 | F | GTGCTGGAAAAGGAATTGGA | 151 | This study |
|  | R | TTTTGCCCTGACATCCCTAC |  |  |
| FN1856 | F | GTTGGACCAGCTCCTGAAAA | 139 | This study |
|  | R | CGTCAACGTGTCCTCCTCTT |  |  |
| FN1857 | F | TGCAATCTGGTGAAATGGAA | 88 | This study |
|  | R | ATTCCTCCCAACCCATAACC |  |  |
| FN1862 | F | ACCTTGGAAGCCAAGTTCCT | 184 | This study |
|  | R | GCTCCACCTGCAATCAAAAT |  |  |
| FN1863 | F | GATGAGTTTGGCGTTCCATT | 182 | This study |
|  | R | TCATGCCATTGCATTTTTGT |  |  |
| FN1866 | F | GCTGGGTCAAGTGATGATGC | 113 | This study |
|  | R | AGAAGTGCATCTCCTCCTGA |  |  |
| FN1867 | F | AGCAGCTCTTGACGTAGCAG | 101 | This study |
|  | R | GCATTCCAGATTTTCCTGCCG |  |  |
| FN1868 | F | TGGCGGCCTTAGCAATAGTT | 142 | This study |
|  | R | TCTCTTCCTAGCTCCTTTGCT |  |  |
| FN1869 | F | TTATGGCACCAGTTTTTGCTG | 149 | This study |
|  | R | TGCCAAAACATCAGCAGCTG |  |  |
| 16S rRNA | F | CAGGATGAACGCTGACAGAA | 122 | This study |
|  | R | GTCCCTAGCTGTGAGGCAAG |  |  |

ORF, open reading frame; F, forward; R, reverse.
