# Supplementary material for: Fusobacterium nucleatum Metabolically Integrates Commensals and Pathogens in Oral Biofilms
Source: mSystems. 2022 Jul 19;7(4):e00170-22. doi: 10.1128/msystems.00170-22 (PMC9426547; doi:10.1128/msystems.00170-22)
Supplement: TABLE S2 [file msystems.00170-22-s0005.docx]

**Table S2.** Characteristics of study participants

| Characteristics | Total subjects | No periodontitis | Mild/moderate periodontitis | Severe periodontitis |
| --- | --- | --- | --- | --- |
| *n* | 102 | 34 | 56 | 12 |
| Age (years) | 37.78 (11.85) | 31.38 (8.33) | 39.02 (11.19) | 50.16 (12.39) |
| Female, *n* (%) | 55 (53.9) | 22 (64.7) | 30 (53.5) | 3 (25.0) |
| Number of teeth |  |  |  |  |
| Total | 27.35 (1.72) | 27.53 (1.26) | 27.67 (0.71) | 25.33 (3.84) |
| Decayed | 0.85 (1.47) | 0.76 (1.35) | 0.75 (1.31) | 1.58 (2.27) |
| Missing | 0.51 (1.60) | 0.14 (0.43) | 0.26 (0.70) | 2.66 (3.84) |
| Treated | 8.39 (6.38) | 5.32 (4.95) | 9.44 (6.71) | 12.16 (5.04) |
| Sound | 18.11 (7.13) | 21.44 (5.61) | 17.48 (7.14) | 11.58 (5.88) |
| PISA | 382.48 (273.61) | 243.96 (123.57) | 381.98 (229.12) | 777.29 (386.97) |

Values are presented as the mean (SD), unless otherwise indicated.
