# Supplementary material for: Fusobacterium nucleatum Metabolically Integrates Commensals and Pathogens in Oral Biofilms
Source: mSystems. 2022 Jul 19;7(4):e00170-22. doi: 10.1128/msystems.00170-22 (PMC9426547; doi:10.1128/msystems.00170-22)
Supplement: TABLE S3 [file msystems.00170-22-s0006.docx]

**Table S3.** Primers and probes used for analysis of plaque samples

| Target | Type | Sequence (5′-3′) | Source |
| --- | --- | --- | --- |
| Universal | F | TCCTACGGGAGGCAGCAGT | (1) |
|  | R | GGACTACCAGGGTATCTAATCCTGTT |  |
|  | Probe | 56-FAM/CGTATTACC/ZEN/GCGGCTGCTGGCAC/3IABkFQ |  |
| *F. nucleatum*  FN0501 | F | TGAAGCTCATGGACCACATTTA | This study |
|  | R | GAGACATTTGAGTCATAGCACCT |  |
|  | Probe | 56-FAM/TCAGCTGTT/ZEN/GATGCTGGAGCAGAT/3IABkFQ |  |
| *S. gordonii*  *arcD* | F | CAATGCTCTTTGCCTTTATGGG | This study |
|  | R | TGATAACTTGGATACCACGCG |  |
|  | Probe | 56-FAM/ACTTGCTCA/Zen/GTGTTGCCCTTATCGT/3IABkFQ |  |
| *P. gingivalis*  16S rRNA | F | ACCTTACCCGGGATTGAAATG | (2) |
|  | R | CAACCATGCAGCACCTACATAGAA |  |
|  | Probe | 56-FAM/ATGACTGAT/Zen/GGTGAAAACCGTCTTCCCTTC/3IABkFQ |  |

F, forward primer; R, reverse primer.

**Supplemental References**

1. Nadkarni MA, Martin FE, Jacques NA, Hunter N. 2002. Determination of bacterial load by real-time PCR using a broad-range (universal) probe and primers set. Microbiology 148:257-266.
2. Kuboniwa M, Amano A, Kimura KR, Sekine S, Kato S, Yamamoto Y, Okahashi N, Iida T, Shizukuishi S. 2004. Quantitative detection of periodontal pathogens using real-time polymerase chain reaction with TaqMan probes. Oral Microbiol Immunol 19:168-176.
